# Modulation of Mood States as a Major Factor in Relapse to Substance Use

**DOI:** 10.3389/fnmol.2012.00081

**Published:** 2012-07-23

**Authors:** Gal Yadid, Lior Redlus, Royi Barnea, Ravid Doron

**Affiliations:** ^1^The Neuropsychopharmacology Lab, The Leslie and Susan Gonda (Goldschmied) Multidisciplinary Brain Research Center, Bar-Ilan UniversityRamat Gan, Israel; ^2^The Neuropsychopharmacology Lab, The Mina and Everard Goodman Faculty of Life Sciences, Bar-Ilan UniversityRamat Gan, Israel; ^3^Department of Education and Psychology, The Open UniversityRa'anana, Israel; ^4^School of Health and Life Sciences, Hadassah Academic CollegeJerusalem, Israel

Substance dependence is characterized by compulsive substance seeking and high vulnerability to relapse. A major challenge in current substance addiction research is not only to understand the immediate effects of substances of abuse on brain operations. It is also to define, at the behavioral and neural levels, how cognitive, emotional, and motivational processes interact with substance use in order to lead to this psychopathological state which defines addiction. For the last decade, research and progress into the biological basis of the addictive process has led to a rapidly growing number of pharmacological agents used to interrupt the progress of the addiction pattern, however without a significant/adequate impact.

It seems that the abstinent versus satiated states differ significantly (Kalivas and Volkow, 2005). Prolonged abstinence from substances of abuse is characterized by dysphoria, depression, and anxiety, coupled with high stress and craving; therefore strongly affecting the quality of life. It is speculated that memories of habitual substance use, produced by anxious and/or stressful emotional states, may have implications for understanding the role of learning and memory processes in substance addiction (Perrine et al., 2008; Packard, 2009). Substance dependent individuals, during their withdrawal, commonly employ thought-suppression to cope with stress and intrusive cognitions about the substance (Garland et al., 2012). Hence, abstinence-induced stress-related mood disorders are considered to be the main valence to define addiction as a chronic brain disorder, and stress is one of the major factors in substance seeking and relapse to its usage (Lu et al., 2003; Koob and Zorrilla, 2010).

Understanding the neurobiological basis of the abstinent state is a necessity to adequately treat substance relapse. The development of addiction and vulnerability to relapse following withdrawal is proposed to be the result of neuroadaptive processes within the central nervous system, leading to impairment in the mechanisms that mediate positive reinforcement and the emergence of affective changes (Weiss et al., 2001). A plethora of gene changes develop in the brain during chronic use or abstinence, which are related to the glutamate/corticoids, CREB/ERK, and NfκB pathways (Nestler, 2005; Li et al., 2008). Regardless the substance, a specific set of genes (Adora2a, Cnr1, Drd1, GPR88, Pde10a, Arpp21, Fam40b, Hpca, and Bc111b; mostly belonging to a huntingtin-centered pathway) were downregulated in the abstinent brain (Kalivas and Volkow, 2005; Le Merrer et al., 2012), hence possibly contribute to the negative affect characterizing protracted abstinence. Not surprisingly, these neuroadaptations, which occur during the addiction process, have been associated with multiple neuropsychiatric disorders (de Lecea et al., 2012).

Chronic stress increases the risk of depression, and is well known to increase relapse to drug seeking behavior (Bruchas et al., 2010). Depressive symptoms were suggested to be associated with abstinence-induced alterations in response to negative distracters (Froeliger et al., 2012). Findings suggest that the severity of depression symptoms are an important predictor of psychosocial treatment efficacy for cocaine dependence and, hence, underline the importance of adequately addressing depression symptoms to improve treatment outcomes (Stulz et al., 2011).

Serotonergic dysregulation in depression and addiction comorbidity was suggested as a novel target for the treatment of addiction and the prevention of drug relapse (Kirby et al., 2011). A few randomized clinical trials support the use of some antidepressant medications for combined cocaine dependence and depression (Rounsaville, 2004). Nonetheless, at the current stage of evidence, data do not unambiguously support the efficacy of antidepressants in the treatment of substance abuse/dependence (Pani et al., 2011). Notably, most negative results came from studies that evaluated selective serotonin reuptake inhibitors (SSRIs), while most positive results were found using norepinephrine/dopamine-reuptake-inhibitors, such as desipramine or bupropion. Although psychiatric symptoms are the prime motive of addicts requesting treatment, they are not always the expression of an associated mental disorder. Indeed, the presence of depressive/anxious symptomatology in the clinical presentation appears to be unnecessarily related to “dual diagnosis” (i.e., addiction and a mental illness). High-frequency abusers demonstrate an associated increased hypothalamic-pituitary-adrenal (HPA) axis activity, a characteristic stress response, to drug-cue exposure (Koob and Zorrilla, 2010). The role of the norepinephrine system in stress is well known, and its involvement in the mechanisms/potentiation of substance abuse has been explored (Belujon and Grace, 2011). Therefore, we suggest that noradrenergic antidepressants are effective in the treatment of substance relapse, since they initially control the stress circuit, and secondarily ease the depressive symptoms.

There is a preponderance of evidence that abuse of substances is parallel with stress disorders (Lu et al., 2003; Koob and Zorrilla, 2010; Sinha, 2011; Moeller, 2012), and anxiogenic effects of abstinence from substances of abuse is dependent on the period of the withdrawal. The administration of anxiolytic agents, such as propranolol and buspirone or the 5-HT3 receptor antagonist ondansetron, after an abstinence period, reversed the anxiogenic effect induced by nicotine, alcohol, cocaine, and opiates (Costall and Naylor, 1992; de Oliveira Citó Mdo et al., 2012).

Several neuropeptides, including corticotrophin-releasing hormone, neuropeptide Y, hypocretin/orexin, nociceptin/orphanin have been shown to be stress-related. Not surprisingly, they also play a pivotal role in addiction, mediating the negative affect associated with stress (Boutrel and de Lecea, 2008; Bruijnzeel, 2012). Endogenous opioid-neuropeptides, such as β-endorphin, dynorphin, enkephalin, and others, have been shown to play a major role in substance reinforcement (Tang et al., 2005; Roth-Deri et al., 2008; Merenlender-Wagner et al., 2009; Wee and Koob, 2010).

Herein we wish to present a new piece to this puzzle, suggesting two putative candidates: the opioid neuropeptide β-endorphin and the neurosteroid dehydroepiandrosterone (DHEA), both shown to modulate mood and addiction.

β-endorphin is an endogenous opioid produced mainly in the arcuate nucleus of the hypothalamus, and released, in part, in the nucleus accumbens (NAc). β-endorphin induces euphoria and has rewarding and reinforcing properties (Roth-Deri et al., 2008). Consequently, it was demonstrated that cocaine induces dopamine-1-receptor dependent β-endorphin release in the NAc (Roth-Deri et al., 2008). Moreover, mice lacking β-endorphin demonstrated attenuation in cocaine-induced conditioned place preference (Marquez et al., 2008). β-endorphin binds with high affinity to μ- and δ-opioid receptors, while its affinity to the κ-opioid receptor is lower (Akil et al., 1984).

Using opioid receptor antagonists, several studies support a role for the μ-opioid type receptor in cocaine addiction in humans (Ghitza et al., 2010) and animal models (Kreek et al., 2009; Simmons and Self, 2009). Some studies showed that extended access to cocaine self-administration was associated with increased activity of the κ-opioid system or its endogenous agonist, dynorphin, in rats (Wee and Koob, 2010). κ-opioid receptor activation decreases acquisition or maintenance of cocaine, ethanol, morphine, and heroin by lowering their reinforcing/rewarding effects (Xi et al., 1998; Logrip et al., 2008; Wee et al., 2009). However, during lower sub-threshold doses of cocaine and morphine during maintenance, or abstinence following long access to cocaine, κ-opioid receptor activation facilitates substance self-administration or reinstatement, possibly through aversive-like and stress-like effects (Kuzmin et al., 1997; Wee and Koob, 2010). There is also some evidence for the involvement of the δ-opioid receptor in reward and addiction, but the studies are equivocal. Some have shown that non-peptidic δ-opioid receptor agonists elicit reward (Longoni et al., 1998), but some reported negligible abuse-related effects (Negus et al., 1998). The selective δ-opioid receptor antagonist naltrindole decreased responding for cocaine in rats, regardless of the schedule of reinforcement (Reid et al., 1995). Conversely, others (de Vries et al., 1995) reported that only a high dose of naltrindole, which decreased locomotor activity, attenuated cocaine self-administration. Intra-accumbal infusion of this δ-opioid receptor antagonist decreased cocaine self-administration, while administration into the VTA significantly increased cocaine-maintained responding (Ward and Roberts, 2007). Some demonstrated that withdrawal from cocaine, resulted in increased anxiety and depression, accompanied the desensitization of δ-opioid receptor function. Furthermore, cocaine-induced anxiety- and depressive-like behaviors were reversed by the δ-opioid receptor agonist SNC80 (Perrine et al., 2008).

Previous studies reported an attenuated β-endorphin response during ethanol or nicotine relapse. One study examined the extent to which β-endorphin response to stress is associated with early smoking relapse. The authors found that smokers who relapsed exhibited attenuated β-endorphin response to stressors, compared to those who maintained abstinence over the same period (Shaw and al’Absi, 2008). Moreover, smokers who underwent weekly exercise sessions had higher β-endorphin plasma levels and demonstrated a reduced smoking rate (Leelarungrayub et al., 2010). In another study, withdrawal from ethanol consumption led to decreased β-endorphin plasma levels. Chronic treatment with acamprosate, which increases β-endorphin plasma concentrations, caused a significant reduction in ethanol intake (Zalewska-Kaszubska et al., 2008).

**Figure 1:**
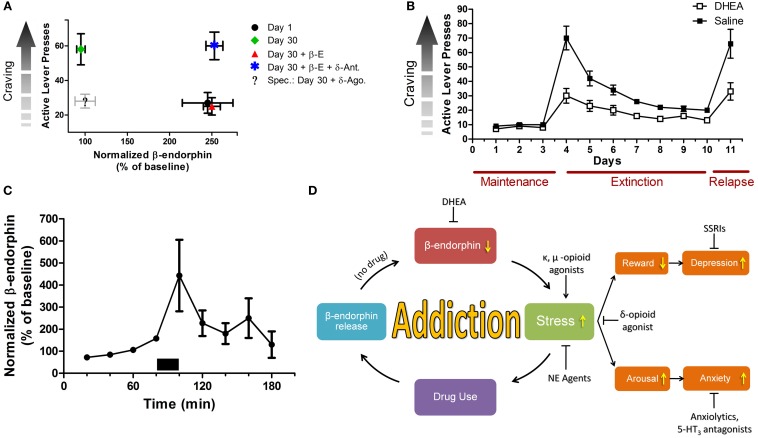
**Stress and mood in relation to substance addiction**. **(A)** β-endorphin levels are inversely correlated with craving during prolonged abstinence. Rats were trained to self administer cocaine (0.75 mg/kg/infusion; 6 h/day; 10 days). Cocaine craving was measured by the number of active lever presses. β-endorphin levels were measured in the extracellular space of the n. accumbens, using the microdialysis technique. Craving was examined on day 1 or on day 30 of forced abstinence. Heightened craving is correlated with low β-endorphin release or low activity of the δ-opioid receptor in the n. accumbens. On withdrawal day 1 (black dot), β-endorphin levels are high and craving is low. On withdrawal day 30 (green rhomb), β-endorphin levels are low and craving is high. Adding β-endorphin (red triangle) markedly reduces craving, while a δ-opioid receptor antagonist (blue star) prevents the β-endorphin effect. Administrating a δ-opioid receptor agonist alone at 30-day abstinence is also expected to markedly reduce craving (legend: “Spec.”). Note: β-E, β-endorphin; δ-Ant, δ-opioid antagonist; δ-Ago., δ-opioid agonist. **(B)** Effect of DHEA treatment or saline on extinction of cocaine self-administration. Rats were trained to self administer cocaine (1.5 mg/kg/infusion), after reaching stable maintenance, rats were either injected with DHEA or saline 90 min prior to placement in the operant chambers. After reaching abstinence, rats were reinstated with 10 mg/kg i.p. cocaine. DHEA significantly facilitates withdrawal and attenuates drug-induced relapse. **(C)** DHEA and β-endorphin are linked together. Using the reverse microdialysis technique, DHEA-sulfate (30 nM, marked by a bar) was applied into the n. accumbens, and β-endorphin levels in dialysates were measured. Perfusion of DHEA-sulfate causes a significant *in situ* transient increase in β-endorphin release. **(D)** A flow chart summarizing the role of stress and mood states in addiction. Note: stress is a core reinforcing component in addiction, and a major modulator of mood states. Hence, stress modulators that secondarily affect mood, strongly oppose relapse to substance usage. NE, norepinephrine; SSRIs, selective serotonin reuptake inhibitors.

Preliminary results elucidate a novel role for β-endorphin in incubation of cocaine craving, a model that evaluates abuse-related drug effects a long time after forced abstinence (Figure 1A). During the satiated state, β-endorphin levels correspond to substance self-administration. Associated cues throughout short-term withdrawal trigger elevated β-endorphin release (Roth-Deri et al., 2008). Throughout long-term abstinence, cues are unable to elicit enough β-endorphin release, concurrently with drug craving. Interestingly exogenous β-endorphin negated the heightened craving during long-term abstinence by acting on the δ-opioid-like receptor (unpublished results). Therefore, restoring abstinence-induced deficits in β-endorphin levels may be an important factor in preventing craving and maintaining abstinence.

We suggest that anxious-like and impulsive responses are linked to the compulsive seeking behavior observed after abstinence from substance abuse, through the δ-opioid receptor. By applying δ-opioid receptor agonists, we may bypass the lower efficacy of opioid-like receptor activity in the abstinence phase. Accordingly, this could cause a decrease in craving, simultaneously with decreased anxiety-like and depressive-like behaviors during extinction response.

Another candidate for intervention in substance abuse and relapse is DHEA, a natural steroid produced from cholesterol by the adrenal glands. DHEA is also produced in the gonads, adipose tissue, and the brain. It is structurally similar to, and is a precursor of, androstenedione, testosterone, and estrogens (Yadid et al., 2010).

Studies indicate that DHEA administration improves memory and cognitive processing; acts as a growth hormone in helping neurons grow new dendrites and controls levels of the stress hormone cortisol (Flood et al., 1988; Ulmann et al., 2009; Yadid et al., 2010). In healthy men and women, it was found that DHEA supplementation improved mood and sense of well being, including better quality of sleep, increased energy, relaxation, and higher capability of handling stress (Morales et al., 1994). Long-term treatment has been shown to modulate distress, improve mood, and relieve depressive-like symptoms (Wolkowitz and Reus, 1999).

Recently it was successfully shown in an animal model of addiction that chronic exposure to exogenous DHEA markedly attenuated cocaine self-administration and decreased cocaine-seeking behavior of rats when applied during cocaine intake (Doron et al., 2006a) or during abstinence (Figure 1B). In another two preclinical studies it was found that DHEA attenuated reinstatement of cocaine-seeking behavior in rats (Doron et al., 2006b; Malkesman et al., 2006) and significantly increased neurogenesis (generation of newly formed neurons; Charalampopoulos et al., 2008; Yadid et al., 2010). In addition, the effect of DHEA was mimicked in heroin addicted rats (unpublished data) and humans (Maayan et al., 2008).

Interestingly, DHEA and β-endorphin are linked together. Application of exogenous DHEA-S (phosphorylated DHEA) into the NAc increases extracellular levels of β-endorphin (Figure 1C). Therefore, a lingering decease in brain DHEA supply may decrease extracellular β-endorphin levels, and this may signal lower capacity to cope with mood fluctuations and stress, that accompany the increase of substance craving.

These suggested candidates may represent a hallmark of drug abstinence and an adaptive mechanism in coping with a history of substance abuse disorders (Figure 1D). Prospective prolongation of DHEA and β-endorphin function may result in protracted drug withdrawal.
